# Long-COVID Symptoms in Individuals Infected with Different SARS-CoV-2 Variants of Concern: A Systematic Review of the Literature

**DOI:** 10.3390/v14122629

**Published:** 2022-11-25

**Authors:** César Fernández-de-las-Peñas, Kin Israel Notarte, Princess Juneire Peligro, Jacqueline Veronica Velasco, Miguel Joaquín Ocampo, Brandon Michael Henry, Lars Arendt-Nielsen, Juan Torres-Macho, Gustavo Plaza-Manzano

**Affiliations:** 1 Department of Physical Therapy, Occupational Therapy, Physical Medicine and Rehabilitation, Universidad Rey Juan Carlos, 28933 Madrid, Spain; 2 Department of Health Science and Technology, Faculty of Medicine, Center for Neuroplasticity and Pain (CNAP), Sensory Motor Interaction (SMI), Aalborg University, 9220 Aalborg, Denmark; 3 Department of Pathology, Johns Hopkins University School of Medicine, Baltimore, MD 21205, USA; 4 Faculty of Medicine and Surgery, University of Santo Tomas, Manila 1008, Philippines; 5 Clinical Laboratory, Division of Nephrology and Hypertension, Cincinnati Children’s Hospital Medical Center, Cincinnati, OH 45229, USA; 6 Department of Medical Gastroenterology, Mech-Sense, Aalborg University Hospital, 9000 Aalborg, Denmark; 7 Department of Internal Medicine, Hospital Universitario Infanta Leonor-Virgen de la Torre, 28031 Madrid, Spain; 8 Department of Medicine, School of Medicine, Universidad Complutense de Madrid, 28040 Madrid, Spain; 9 Department of Physical Therapy, Universidad Complutense de Madrid, 28040 Madrid, Spain

**Keywords:** post-COVID-19, long-COVID, delta, alpha, Wuhan, systematic review

## Abstract

The association of SARS-CoV-2 variants with long-COVID symptoms is still scarce, but new data are appearing at a fast pace. This systematic review compares the prevalence of long-COVID symptoms according to relevant SARS-CoV-2 variants in COVID-19 survivors. The MEDLINE, CINAHL, PubMed, EMBASE and Web of Science databases, as well as the medRxiv and bioRxiv preprint servers, were searched up to 25 October 2022. Case-control and cohort studies analyzing the presence of post-COVID symptoms appearing after an acute SARS-CoV-2 infection by the Alpha (B.1.1.7), Delta (B.1.617.2) or Omicron (B.1.1.529/BA.1) variants were included. Methodological quality was assessed using the Newcastle–Ottawa Scale. From 430 studies identified, 5 peer-reviewed studies and 1 preprint met the inclusion criteria. The sample included 355 patients infected with the historical variant, 512 infected with the Alpha variant, 41,563 infected with the Delta variant, and 57,616 infected with the Omicron variant. The methodological quality of all studies was high. The prevalence of long-COVID was higher in individuals infected with the historical variant (50%) compared to those infected with the Alpha, Delta or Omicron variants. It seems that the prevalence of long-COVID in individuals infected with the Omicron variant is the smallest, but current data are heterogeneous, and long-term data have, at this stage, an obviously shorter follow-up compared with the earlier variants. Fatigue is the most prevalent long-COVID symptom in all SARS-CoV-2 variants, but pain is likewise prevalent. The available data suggest that the infection with the Omicron variant results in fewer long-COVID symptoms compared to previous variants; however, the small number of studies and the lack of the control of cofounders, e.g., reinfections or vaccine status, in some studies limit the generality of the results. It appears that individuals infected with the historical variant are more likely to develop long-COVID symptomatology.

## 1. Introduction

The massive spread of the Severe Acute Respiratory Syndrome Coronavirus 2 (SARS-CoV-2), the causative agent of the coronavirus disease 2019 (COVID-19), favored the development of mutations paving the way for several variants to emerge [1]. Among all SARS-CoV-2 variants identified, Alpha (B.1.1.7), Delta (B.1.617.2) and Omicron (B.1.1.529/BA.1) have been considered worldwide as variants of concern (VOCs), in addition to the historical (20A.EU2) variant that originated in Wuhan, China [2]. Several differences, e.g., more viral load, higher transmissibility or potential escape to vaccines, among the VOCs have been described [3]. For instance, the Delta variant exhibits a higher viral load than the historical or Alpha variants [4], whereas the Omicron variant shows the highest level of transmissibility [5]. Monitoring clinical manifestations of SARS-CoV-2 variants could be relevant for the identification, management or control of the pandemic. Preliminary evidence suggests that the associated-onset symptoms and severity of COVID-19 conditions differ among SARS-CoV-2 variants [6,7].

Today, it seems clear that many individuals who survived the SARS-CoV-2 infection develop long-lasting symptoms after the acute phase, termed long-COVID [8] or post-COVID-19 condition [9]. The current data support that almost 60% of COVID-19 survivors could experience long-COVID symptoms during the first year after the infection [10,11], and up to 42% can experience symptoms two years after [12]. The presence of long-COVID is associated with worse health-related quality of life [13]. It is obvious that most studies published at this timepoint investigated the presence of long-COVID symptoms in individuals infected during the first wave of the pandemic, when the historical variant was predominant [10,11,12].

The widespread of the Omicron variant, due to its higher transmissibility, provoked a massive increase in the number of contagions in 2022, leading to an exponential increase in people at risk of experiencing long-COVID. For instance, the United Kingdom Office for National Statistics estimated that the number of individuals experiencing long-COVID increased from 1.3 million in January 2022 to 2 million on 1 May 2022 [14]. Other articles suggested that the risk of developing long-COVID is lower with the Omicron variant than with other VOCs [15,16]. Accordingly, since millions of people will experience long-COVID [17], the identification of an association of long-COVID with the SARS-CoV-2 variants is needed. Systematic reviews on mechanisms [18], prognostic factors [19] or the effect of vaccines on long-COVID [20] were previously published; however, no review to date has systematically investigated differences in long-COVID symptomatology depending on the SARS-COV-2 variant. Thus, the present review aims to answer the following research questions: (1) what is the prevalence of long-COVID symptoms in people infected with different SARS-CoV-2 variants, and (2) is there any difference in terms of long-COVID symptoms among these variants?

## 2. Methods

A systematic review investigating post-COVID symptoms depending on the SARS-CoV-2 variant of concern according to the Preferred Reporting Items for Systematic Reviews and Meta-Analyses (PRISMA) statement of 2020 was conducted [21]. The review study was registered in the Open Science Framework (OSF) database (https://osf.io/nk5rb).

### 2.1. Search Strategy

An electronic search for articles published up to 25 October 2022 on the MEDLINE, CINAHL, PubMed, EMBASE and Web of Science databases, as well as on the preprint servers medRxiv and bioRxiv, was conducted by two different authors using the following search terms: “long-COVID” OR “post-acute COVID” OR “post-COVID-19 condition” OR “long hauler” AND “variant” OR “Wuhan” OR “historical” OR “Alpha” OR “Delta” OR “Omicron” OR “20A.EU2” OR “B.1.1.7” OR “B.1.617.2” or “B.1.1.529/BA.1”. The combinations of these search terms using Boolean operators are outlined in Table 1.

### 2.2. Selection Criteria

The inclusion and exclusion criteria were described according to the Population, Intervention, Comparison, and Outcome (PICO) principle:

*Population:* Adults (>18 years) previously infected with SARS-CoV-2 and diagnosed with real-time reverse transcription-polymerase chain reaction (RT-PCR) assay or serological test. Subjects could have been hospitalized or not by SARS-CoV-2 acute infection. One group should have been infected with Alpha (B.1.1.7), Delta (B.1.617.2) or Omicron (B.1.1.529/BA.1) variants. We included studies defining the particular variant of concern based on genomic sequencing or the time period of predominance in each particular country.
*Intervention:* Not applicable.
*Comparison:* Articles should investigate the presence of long-COVID symptoms in at least one SARS-CoV-2 variant of concern that is different from the historical strain.
*Outcome:* Collection of long-COVID symptoms developed after SARS-CoV-2 infection by personal, telephone or electronic interviews. We considered any long-COVID symptom, e.g., fatigue, dyspnea, pain, brain fog, memory loss, skin rashes, palpitations, cough, and sleep problems. We included all studies regardless of the definition of long-COVID used.

### 2.3. Screening Process, Study Selection and Data Extraction

Observational cohort (retrospective/prospective), cross-sectional, and case-control studies describing the presence of symptoms after an acute SARS-CoV-2 infection with at least one VOC different from the historical variant were included. This review was limited to human studies and English language papers. Editorials or opinion articles without data were excluded. Research letters or correspondence showing new data were included.

Two authors screened all of the titles and abstracts of the publications obtained from the database search and removed duplicates. The full text of eligible articles was retrieved and analyzed. The following data were extracted from each study: authors, country, design, sample size, setting, long-COVID definition, differences in long-COVID among variants, and follow-up periods. Discrepancies between authors in any part of the screening and data extraction process were resolved by a third author, if necessary.

### 2.4. Methodological Quality

The Newcastle–Ottawa Scale (NOS) was applied independently by two authors to evaluate the methodological quality of the studies. The NOS is a nine-star rating system evaluating the risk of bias of case-control and cohort studies [22]. In cohort studies, the NOS evaluates: case selection (i.e., representativeness of the cohort, selection of the non-exposed cohort, case definition, outcome of interest), comparability (i.e., proper control for age, sex or other factors, between-group comparisons) and exposure (i.e., outcome assessment, enough follow-up, adequate follow-up). In case-control studies, the NOS is adapted. For instance, the case selection item includes adequate case definition or controls selection. The quality of longitudinal cohort studies or case-control studies is classified as: high quality (seven–nine stars), medium quality (five–six stars) or low quality (<four stars). In cross-sectional cohort studies, a maximum of three stars can be awarded: good quality (three stars), fair quality (two stars) or poor quality (one star). Methodological quality was initially evaluated by two authors. If there was disagreement, a third researcher arbitrated the final decision.

### 2.5. Data Synthesis

A meta-analysis was not deemed to be appropriate due to the high heterogeneity between studies, particularly the inclusion of different follow-up periods and settings. Accordingly, we conducted a synthesis of the data by addressing the population, post-COVID symptoms by SARS-CoV-2 variant, limitations, and methodological quality of the studies.

## 3. Results

### 3.1. Study Selection

**Figure 1:**
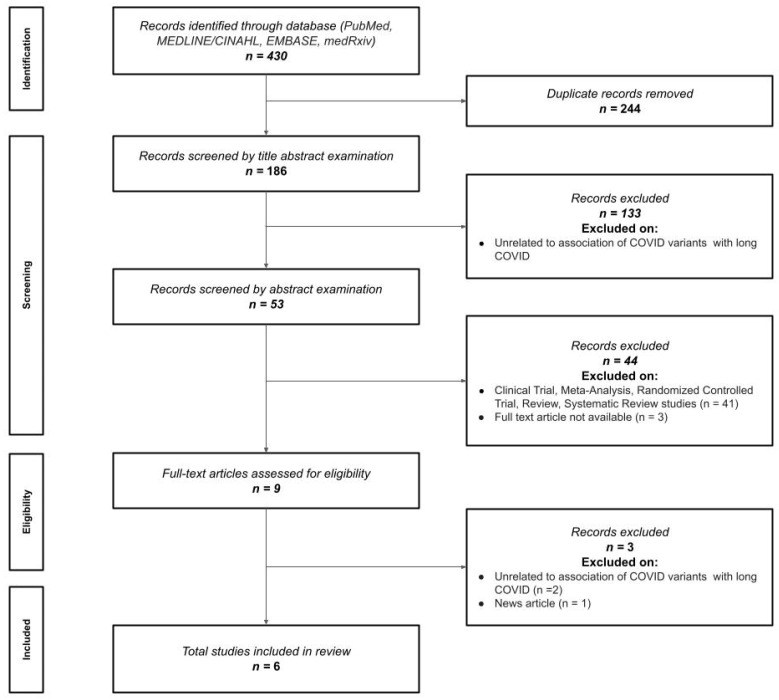
Preferred Reporting Items for Systematic Reviews and Meta-Analyses (PRISMA) flow diagram. *n*: number of studies.

The electronic search identified 430 potential titles for screening. After removing duplicates and papers not directly related to SARS-CoV-2 variants, 186 studies remained for title/abstract examination. A total of 133 (*n* = 133) were excluded after title examination, and another 53 were excluded after abstract examination, leading to 9 articles for the full review. A total of six articles, five peer-reviewed [23,24,25,26,27] and one preprint [28], were finally included (Figure 1).

### 3.2. Sample Characteristics

The characteristics of the populations of the included studies are shown in Table 2. The total sample consisted of 100,832 COVID-19 survivors (56.6% female). Based on five studies reporting participant age [23,24,25,26,27], the mean age of the sample was 50.1 years. One paper [28] did not report the mean age of its sample. Two studies [24,26] exclusively included hospitalized patients (*n* = 667, 51.1% female; mean age: 59.2 years) whereas one study [25] exclusively included non-hospitalized patients (*n* = 739, 74.5% female; mean age: 42.7 years). The remaining papers did not provide data about hospitalization [23,27,28].

Two studies [25,26] included patients infected with the historical/Wuhan (*n* = 355) variant and patients infected with the Alpha (*n* = 512) variant. Two studies [23,26] included patients infected with the Delta (*n* = 41,563) variant, while four studies [23,24,27,28] included patients infected with the Omicron (*n* = 57,616) variant. In one study [25], the sample included patients infected with the Delta or Omicron variants (*n* = 284), without distinction. All studies defined the particular variant of concern based on the time period of predominance in each particular country, except one [26] which confirmed the SARS-CoV-2 variant by genomic sequencing.

The follow-up period collecting the prevalence of post-COVID symptoms was highly heterogeneous, ranging from one [23] to two [27], three [24] or six [26] months after the infection. Two studies did not specify the time from SARS-CoV-2 infection [25,28].

### 3.3. Methodological Quality

Three cross-sectional cohort [24,26,28], one longitudinal observational cohort [25], one retrospective cohort [27], and one case-control study [23] were included. All six studies were of high methodological quality [23,24,25,26,27,28]. No disagreement between authors was found. Table 3 presents the NOS score for each study and a summary of every item.

### 3.4. Long-COVID Symptoms by SARS-CoV-2 Variant

The presentation of the results was heterogeneous, since three studies provided crude prevalence data [24,27,28], two provided an odds ratio [23,25], and the remaining one provided the number of long-COVID symptoms [26].

Three studies [24,26,27] used the definition of post-COVID-19 condition proposed by Soriano et al. [9]: “post-COVID-19 condition occurs in subjects with positive history of probable or confirmed SARS-CoV-2 infection, usually 3 months from onset of COVID-19, with symptoms that last for at least 2 months and cannot be explained by alternative diagnosis”. The remaining studies [23,25,28] used the definition proposed by the National Institute for Health and Care Excellence (NICE) [29]: “long-COVID consists of signs and symptoms developed during or following a disease consistent with COVID-19 and which continue for more than four weeks but they are not explained by alternative diagnoses”.

Azzolini et al. observed a prevalence of long-COVID symptoms of 48.1% (95% CI 39.9–56.2%) with the historical variant, 35.9% (95% CI 30.5–41.6%) with the Alpha variant, and 16.5% (95% CI 12.4–21.4%) with a mix of the Delta and Omicron variants; however, the multivariate analysis did not reveal a significant association among variants [25]. Fernández-de-las-Peñas et al. reported that previously hospitalized patients infected with the historical variant exhibited a greater number of long-COVID symptoms than those infected with the Alpha or Delta variants [26]. The prevalence of long-COVID symptoms in people infected with the Omicron variant ranged from 5%, as reported by Morioka et al., [24] to 25%, as reported by Qasmieh et al. [28]. It should be noted that the sample included in the study by Morioka et al. was extremely small (*n* = 54), and just one individual infected with Omicron exhibited long-COVID.

Only two studies [26,27] detailed long-COVID symptoms. These studies found that fatigue was the most prevalent long-COVID symptom, regardless of the SARS-CoV-2 variant. Other symptoms reported in the reviewed studies included pain, one of the most self-perceived bothersome post-COVID symptoms, [26] and other less bothersome symptoms such as cough [27].

## 4. Discussion

This systematic review explored the prevalence of long-COVID depending on the SARS-CoV-2 variant. The results suggest that individuals infected with the Omicron variant are at a lower risk of developing long-COVID symptoms; however, the results should be considered with caution because most studies did not control other confounding factors, e.g., reinfections or vaccine status. All studies were of high methodological quality but also showed high heterogeneity. The most prevalent long-COVID symptom, which was common to all SARS-CoV-2 variants, was fatigue.

Previous meta-analyses that pooled the prevalence data of long-COVID reported that 40–60% of individuals infected during the first wave of the pandemic, with the historical variant, can develop long-COVID symptomatology up to two years after infection [10,11,12]. The present review is the first to systematically evaluate the prevalence of long-COVID by SARS-CoV-2 variants of concern. The current review found prevalence rates lower than 25% in people infected with the Omicron variant [24,28]. Although not directly, comparing the data from the current review with the prevalence rates from published meta-analyses analyzing studies including patients infected with the historical variant reaching 60% of the patients [10,11,12], it could be argued that infections with the Omicron variant could result to lower risk of developing long-COVID symptomatology than infections with the Delta [25,27] or other previous [23,24] variants. In other words, patients infected with the historical variant during the first wave of the COVID-19 pandemic would be at a higher risk of developing long-COVID than those infected by a subsequent variant. However, this assumption should be considered with caution at this stage because of the small number of studies and the lack of control of other cofounders, e.g., reinfections or vaccine status, in some studies. In addition, studies investigating long-COVID symptoms in individuals infected with the traditional or Alpha variants [26] were conducted in hospitalized patients. Therefore, the overrepresentation of chronic fatigue can be present in hospitalized cases and may also be associated with post-intensive care syndrome and/or treatments received at the hospital. This situation can also be applied to current meta-analyses [10,11,12], although emerging evidence suggests that non-hospitalized patients exhibit high prevalence rates of long-COVID symptomatology up to two years after infection [30]. Nevertheless, it should be noted that no study included a control group with individuals not infected by SARS-CoV-2.

The results identified that fatigue was the most prevalent post-COVID symptom, regardless of the SARS-CoV-2 variant, confirming the assumption that coronavirus epidemic left survivors with post-infection fatigue [31]. These findings confirm that post-COVID fatigue will represent a challenge for healthcare professionals, since long-COVID respiratory symptoms, particularly fatigue or dyspnea, are associated with a higher related burden [32]. In fact, fatigue is seen as a long-lasting post-COVID symptom showing a slow recovery curve during the following years after the infection [33]. The fact that fatigue would be a common post-COVID symptom regardless of the SARS-CoV-2 variant suggests that pathogenic cell-to-cell mechanisms associated with the development of post-COVID fatigue could be similar among SARS-CoV-2 variants, although there are differences in the viral load, transmissibility or potential reinfection among variants. Increasing evidence reveals that individuals with long-COVID share common symptoms with myalgic encephalomyelitis/chronic fatigue syndrome [34] and also share similar underlying mechanisms, i.e., endothelial dysfunction [35]. A better understanding of the mechanisms behind post-COVID fatigue is needed to improve the management of patients with long-COVID, regardless of the SARS-CoV-2 variant.

Several hypotheses were proposed to explain the decline in the presence of long-COVID with subsequent SARS-CoV-2 variants. It is expected that the first time the body fights off a new virus such as SARS-CoV-2, the response would be more erratic, and the possibility of developing severe symptoms is more likely. A potential explanation could be related to the innate nature of subsequent SARS-CoV-2 variants; however, this did not happen with the Delta (B.1.617.2) variant, where a higher viral load was identified [4], leading to the most devastating wave in terms of worldwide deaths. Another explanation could be the presence of immunity developed due to previous infections (pre-existing immunity) [36,37]. Accordingly, it would be expected that the development of post-infection symptoms would be higher with the historical variant. Preliminary data would support this assumption. Fernández-de-las-Peñas et al. reported that individuals infected with the historical variant exhibited a greater number of post-COVID symptoms, particularly respiratory symptoms, e.g., dyspnea, than those patients infected with the Alpha or Delta variant [26]. Since the presence of post-COVID respiratory symptoms is associated with a higher post-COVID burden [31], it is possible that the health and economic burden of long-COVID symptoms caused by the historical SARS-CoV-2 variant would be higher than the burden associated with other variants.

Furthermore, it has been identified that the onset symptoms of the Omicron variant are less specific than the onset symptoms associated with previous SARS-CoV-2 variants, since flu-like symptoms, e.g., sneezing or cough, are more prevalent with Omicron [38], whereas other, more specific COVID-19 symptoms, such as ageusia or anosmia, are more prevalent with previous SARS-CoV-2 variants, e.g., the historical or Delta variants [39]. In fact, a study found that the predominance of the Omicron (B.1.1.529) variant was associated with a remarkably higher number of internet searches for upper respiratory symptoms more associated with the common flu, accompanied by a lower interest for other bothersome COVID-19-associated symptoms e.g., dyspnea [38]. However, the fact that the Omicron variant shares symptoms with the common flu does not mean that COVID-19 should be considered a flu caused by the influenza virus [40].

In addition, worldwide vaccination started at the same time that SARS-CoV-2 variants of concern, e.g., Alpha, were widespread. Accordingly, the prevalence of long-COVID symptoms in people infected by the Alpha, Delta or Omicron variants should be considered under the potential effect of vaccines. In fact, current evidence supports that vaccination before infection decreases the risk of developing long-COVID [41]. Although some studies controlled for the vaccination status in their analyses, their small sample sizes limit the extrapolation of the conclusions. More importantly, no study controlled the effect of reinfections. Therefore, similar to vaccines, where the effect on long-COVID is different depending on receiving one, two or booster doses, people re-infected with different SARS-CoV-2 variants could be at a higher risk of long-COVID development [42].

Finally, we cannot exclude the potential influence of the surrounding factors around each wave that are, hence, associated with each SARS-Co-V-2 variant. For instance, several outbreak-associated factors, e.g., social alarm, somatization, post-traumatic stress disorder, fear or uncertainty about the prognosis, stigmatization, physical inactivity, and lack of exercise during lockdown, were more pronounced during the first wave associated with the historical variant due to its association with a worldwide lockdown compared to subsequent SARS-CoV-2 variants. These surrounding COVID-19 outbreak factors could be more associated with emotional (e.g., anxiety, depression, sleep disorders) rather than physical (e.g., fatigue, dyspnea) or cognitive (e.g., brain fog) symptoms.

The results of this review summarizing the prevalence rates of long-COVID symptoms according to SARS-CoV-2 variant should be considered according to its strengths and limitations. The main strengths were the rigorous methodology applied for the literature search, the study selection, the screening for eligibility and the rigorous methodological quality assessment; however, it should be noted that the NOS has been criticized due to showing small inter-rater reliability [43]. In the current review, both authors were in almost perfect agreement, probably due to the small number of studies. Additionally, some limitations of the review should be also recognized. First, a meta-analysis could not be conducted because of the heterogeneity in the setting and follow-ups among the studies. Second, the number of studies investigating long-COVID symptomatology in individuals infected with SARS-CoV-2 variants different from the historical stain is small. In addition, most studies did not confirm the SARS-CoV-2 variant and only assumed the potential variant based on the date of infection and the predominant variant at that particular time in each country. Additionally, it is impossible with the available data to exclude the possibility that SARS-CoV-2 infection was preceded by some of the self-reported symptoms, such as fatigue and cognitive impairments. Third, no study provided data separately by sex; therefore, sex differences were not analyzed. Finally, the studies were highly heterogenous in the collection of long-COVID symptoms, clinical settings, and follow-ups. Thus, we proposed the use of specific patient-reported outcome measures (PROM), e.g., the long-COVID Symptom and Impact Tool [44], in order to obtain more homogeneous data. Similarly, the use of specific questionnaires evaluating the severity of some symptoms, e.g., fatigue, as well as other aspects, e.g., health-related quality of life, are encouraged in future studies. In summary, the current evidence on long-COVID symptoms by SARS-CoV-2 variants should be considered with caution at this stage. It is needed to control for reinfections and confirm the infected variant with genome sequencing.

## 5. Conclusions

This systematic review summarizes the current evidence on long-COVID symptoms according to SARS-CoV-2 variant. The available evidence suggests that subjects infected with the Omicron variant could be at a lower risk of developing long-COVID symptoms than those infected with other variants; however, the results should be considered with caution because of the small number of studies and the heterogeneity in the collected data. Fatigue seems to be the most prevalent post-COVID symptom in all SARS-CoV-2 variants. The presence of long-COVID regardless of the SARS-CoV-2 variant would support the need for specific management attention. Standardized long-COVID follow-up questionnaires/protocols should also be developed to ensure more homogenous data collection across studies. Overall, the current and previous data suggest that individuals infected with the historical variant are at a higher risk of developing long-COVID symptomatology. 

## Figures and Tables

**Table 1:** Database formulas during the literature search.

PubMed Search Formula #1 “post-acute COVID-19 syndrome” [Supplementary Concept] OR “post-acute COVID-19 syndrome” [All Fields] OR “long-COVID” [All Fields] OR “long-COVID symptoms” [All Fields] OR “long hauler” [All Fields] OR “post-COVID-19” [All Fields] OR “post-acute COVID-19 symptoms” [All Fields] OR “COVID-19 sequelae” [All Fields] #2 “SARS-CoV-2 variants” [Supplementary Concept] OR “alpha” [All Fields] OR “B.1.1.7” [All Fields] OR “beta” [All Fields] OR “B.1.351” [All Fields] OR “gamma” [All Fields] OR “P.1” [All Fields] OR “delta” [All Fields] OR “B.1.617.2” [All Fields] OR “omicron” [All Fields] OR “B.1.1.529” [All Fields] #3 #1 AND #2
Medline/CINAHL (via EBSCO) Search Formula #1 “post-acute COVID-19 syndrome” OR “long-COVID” OR “long-COVID symptoms” OR “long hauler” OR “post-COVID-19” OR “post-acute COVID-19 symptoms” OR “COVID-19 sequelae” #2 “SARS-CoV-2 variants” OR “alpha” OR “B.1.1.7” OR “beta” OR “B.1.351” OR “gamma” OR “P.1” OR “delta” OR “B.1.617.2” OR “omicron” OR “B.1.1.529” #3 #1 AND #2
WOS (EMBASE)/Web of Science Search Formula (“post-acute COVID-19 syndrome” OR “long-COVID” OR “long-COVID symptoms” OR “long hauler” OR “post-COVID-19” OR “post-acute COVID-19 symptoms” OR “COVID-19 sequelae” AND (“SARS-CoV-2 variants” OR “alpha” OR “B.1.1.7” OR “beta” OR “B.1.351” OR “gamma” OR “P.1” OR “delta” OR “B.1.617.2” OR “omicron” OR “B.1.1.529”)
medRxiv (“post-acute COVID-19 syndrome” OR “long-COVID” OR “long-COVID symptoms” OR “long hauler” OR “post-COVID-19” OR “post-acute COVID-19 symptoms” OR “COVID-19 sequelae” AND (“SARS-CoV-2 variants” OR “alpha” OR “B.1.1.7” OR “beta” OR “B.1.351” OR “gamma” OR “P.1” OR “delta” OR “B.1.617.2” OR “omicron” OR “B.1.1.529”)

**Table 2:** Data from studies investigating long-COVID symptoms according to SARS-CoV-2 variants.

Author	Variant	Country Study Period	Design Sample	Age	Symptoms Assessment	Prevalence of Long-COVID by Variant	Long-COVID Definition
Morioka et al., 2022 [24]	Omicron	Japan Omicron (*n* = 53) 1 December 2021–9 February 2022 Follow-up: 3 months after Other variants (*n* = 502) February 2020–November 2021	Cross-sectional *n* = 555 Female *n* = 314 Hospitalized *n* = 53	Omicron age 56 (35–69) Other variants age 48 (42–55)	Telephone interviews Self-reporting questionnaire survey	Omicron group At least one post-COVID symptom 5.6% Other variants group At least one post-COVID symptom 55.6%	Symptoms that persisted for at least 2 months within 3 months of COVID-19 onset
Azzolini et al., 2022 [25]	Historical Alpha Delta–Omicron	Italy March 2020 to April 2022	Longitudinal observational cohort *n* = 739 Female *n* = 551	NR	Survey questionnaire	OR (95% CI) Wave 1 NR Wave 2 0.72 (0.48–1.08) Wave 3 1.34 (0.26–7.01)	At least one symptom with a duration of more than 4 weeks after the infection
Qasmieh et al., 2022 [28]	Omicron	United States June 2022–July 2022	Cross-sectional *n* = 1036 Female *n* = 528 Hospitalized NR	Range 18–65 y	Survey via landlines (IVR) and mobile phones (SMS text)	Prevalence (95% CI) 21.5% (18.2–24.7) Men: 15.5 (11.6–19.4) Women: 27.3 (22.2–32.4) Fully vaccinated 25.1% (16.9–33.4) Not vaccinated 22.2% (16.6–27.9) Boosted 19.2% (14.8–23.5)	Symptoms more than 4 weeks after the start of COVID-19 that are not explained by something else
Antonelli et al., 2022 [23]	Omicron Delta	United Kingdom June 2021–March 2022	Case-control *n* = 97,364 Delta (*n* = 41,361) Omicron (*n* = 56,003) Female *n* = 55,205 Hospitalized NR	53 years	Self-reported data from the COVID Symptom Study app	OR (95% CI) Omicron vs. Delta >6 m post-vaccination 0.26 (0.20–0.32) 3–6 m post-vaccination 0.24 (0.19–0.32) <3 m post-vaccination 0.50 (0.43–0.59)	New or ongoing symptoms 4 weeks or more after acute COVID-19
Arjun et al., 2022 [27]	Omicron	India First week of January–middle of February 2022	Retrospective cohort *n* = 524 Female *n* = 212 Hospitalized *n* = 27	Age Mean (SD) 36 (14);	Telephone interviews	Prevalence (95% CI) 8.2% (6% to 10.9%)	Post-COVID-19 condition defined as signs and symptoms that develop during or after COVID-19, continue for more than 12 weeks and are not explained by an alternative diagnosis
Fernández-de-las-Peñas et al., 2022 [26]	Historical (*n* = 201) Alpha (*n* = 211) Delta (*n* = 202)	Spain March 2020–August 2021	Cross-sectional cohort *n* = 614 Female *n* = 327 Hospitalized *n* = 614	Mean (SD) Historical 60.5 (15.5) Alpha 70.0 (15.5) Delta 56.5 (21.0)	Telephone interviews	Historical variant Number symptoms: 2.7 ± 1.3 Fatigue 68.2% Dyspnea 29.35% Alpha variant Number symptoms: 1.8 ± 1.1 Fatigue 71.5% Dyspnea 13.75% Delta variant Number symptoms: 2.1 ± 1.5 Fatigue 76.35% Dyspnea 12.8%	Development of symptoms 6 months after the acute phase of the infection

NR: not reported.

**Table 3:** Methodological quality (Newcastle–Ottawa Scale—NOS) of studies included in the review.

Study	Selection				Comparability		Exposure			Score
	Adequate Case Definition	Representativeness of Cases	Selection of Controls	Definition of Controls	Controlled for Age	Controlled for Additional Factors	Ascertainment of Exposure	Same Method for Cases and Controls	Non-Response Rate	
Antonelli et al., 2022 [23]	★	★	★	★	★	★	★	★	★	9/9
Study	Selection				Comparability		Outcome			Score
	Representativeness of the exposed cohort	Selection of the non-exposed cohort	Ascertainment of exposure	Outcome of interest was not present at the start of the study	Comparability of cohorts on the basis of the design or analysis		Assessment of outcome	Was the follow-up long enough for outcomes to occur?	Adequacy of the follow-up of cohorts	
					Main factor	Additional factor				
Marioka et al., 2022 [24]	★	★	★		★	★		★	★	7/9
Azzolini et al., 2022 [25]	★	★	★		★	★		★	★	7/9
Qasmieh et al., 2022 [28]	★	★	★		★	★	★	★	★	8/9
Arjun et al., 2022 [27]	★	★	★		★	★		★	★	7/9
Fernández-de-las-Peñas et al., 2022 [26]	★	★	★		★	★	★	★	★	8/9
